# SuperEdgeGO: Edge-supervised graph representation learning for enhanced protein function prediction

**DOI:** 10.1371/journal.pcbi.1013343

**Published:** 2025-08-01

**Authors:** Shugang Zhang, Yuntong Li, Wenjian Ma, Qing Cai, Jing Qin, Xiangpeng Bi, Huasen Jiang, Xiaoyu Huang, Zhiqiang Wei

**Affiliations:** 1 College of Computer Science and Technology, Ocean University of China, Qingdao, China; 2 College of Education, Qingdao Hengxing University of Science and Technology, Qingdao, China; 3 College of Computer Science and Technology, Qingdao University, Qingdao, China; Guangxi University, CHINA

## Abstract

Understanding the functions of proteins is of great importance for deciphering the mechanisms of life activities. To date, there have been over 200 million known proteins, but only 0.2% of them have well-annotated functional terms. By measuring the contacts among residues, proteins can be described as graphs so that the graph leaning approaches can be applied to learn protein representations. However, existing graph-based methods put efforts in enriching the residue node information and did not fully exploit the edge information, which leads to suboptimal representations considering the strong association of residue contacts to protein structures and to the functions. In this article, we propose SuperEdgeGO, which introduces the supervision of edges in protein graphs to learn a better graph representation for protein function prediction. Different from common graph convolution methods that uses edge information in a plain or unsupervised way, we introduce a *supervised* attention to encode the residue contacts *explicitly* into the protein representation. Comprehensive experiments demonstrate that SuperEdgeGO achieves state-of-the-art performance on all three categories of protein functions. Additional ablation analysis further proves the effectiveness of the devised edge supervision strategy. The implementation of edge supervision in SuperEdgeGO resulted in enhanced graph representations for protein function prediction, as demonstrated by its superior performance across all the evaluated categories. This superior performance was confirmed through ablation analysis, which validated the effectiveness of the edge supervision strategy. This strategy has a broad application prospect in the study of protein function and related fields.

## Author summary

Understanding protein functions is vital for biological discovery, yet only 0.2% of known proteins currently have well-annotated functional terms, leaving the vast majority uncharacterized. While computational methods have emerged to bridge this gap, most rely heavily on protein sequence data or underutilize structural information, which limits their accuracy. In this work, we address a key oversight in existing approaches—the insufficient use of interactions between amino acid residues (edges) in protein structures. We introduce SuperEdgeGO, a graph-based method that explicitly supervises these residue interactions during model training. By integrating edge information directly into protein representations through a novel attention mechanism, our approach captures structural features more effectively. Experiments show that SuperEdgeGO outperforms state-of-the-art methods across all functional categories of proteins, offering a more reliable tool for automated function prediction. This advancement could accelerate the annotation of uncharacterized proteins, aiding drug discovery, disease mechanism studies, and broader biological research. Our work highlights the untapped potential of edge-centric modeling in computational biology and opens avenues for similar strategies in related fields.

## Introduction

Proteins are fundamental to life and play a central role in the structure and function of cells. They are composed of amino acids, and the specific amino acid sequence determines their unique three-dimensional structure [1], which is directly related to their biological functions including acting as enzymes to catalyze biochemical reactions, constituting the cytoskeleton, transmitting signals, transporting molecules, and participating in immune responses [2–5]. With the development of high-throughput sequencing technology, more than 200 million sequences are available in protein sequence databases. However, only approximately 0.2% of these sequences are manually labeled due to the huge labor and experimental costs of traditional biological measurements [6], leading to a huge gap between the great number of proteins to be annotated and the limited experimental resources. Therefore, it has become particularly important to develop an efficient computational method to predict protein functions.

To devise an effective protein function prediction method, the most important step is to learn an effective representation of the protein to be predicted, which is termed *protein representation learning*. In the early stage, due to the lack of experimentally resolved structures of proteins, most computational methods relied only on sequences to predict protein functions [7–10]. For example, BLAST generalizes function annotations of known proteins to unknown ones according to the homologous similarity of amino acid sequences [8]. DeepGO [9] and DeepGOA [10] adopt convolutional neural networks (CNN) to handle protein sequences, and the sequential features are combined with some macroscopic semantic features extracted from protein interaction networks or hierarchical graphs of GO terms. Obviously, the major drawback of the above category is that it ignores the structural information of the protein. Since the structure of proteins is critical for them to perform their functions; therefore, the performance of approaches relying solely on sequences is far from satisfactory. Motivated by the above limitation and with the accumulation of experimentally solved protein structures, the structure-based methods gradually emerged, where the three-dimensional protein structure is converted to a *graph* via measuring and thresholding the distance between two Cα atoms of two residues. A pioneering work is DeepFRI [5], which predicts protein functions by applying graph convolutional networks (GCN) to protein graphs converted from the experimentally solved structures. Similar representation techniques have also been applied to other more particular protein function prediction tasks such as drug-target affinity (DTA) [11] and protein-protein interaction (PPI) [12] predictions. The structure-based methodology has seen further advancements since the breakthrough of highly accurate prediction of protein structures by AlphaFold2 [13]. The reliability of AlphaFold2-predicted structures has been evaluated in our previous study, where we found that, for the protein function prediction task, training models with the predicted structural data achieved comparable performance to the one with the corresponding experimentally resolved structures. This important finding has inspired more structure-based research in this area [14–17]. Most recently, Jiao et al. [18] proposed Struct2GO that also utilized AlphaFold2-predicted structures to generate protein graphs, on which GCN and graph hierarchical pooling with self-attention mechanism were used to generate the protein representation. Evaluation results demonstrate a state-of-the-art performance on the benchmark dataset.

Despite the great improvement of structure-based methods over earlier sequence-based methods, most of them tried to improve the model performance in terms of node representation, for example, by introducing myriad residue features. In contrast, from the perspective of structure, only some basic graph convolutional network (GCN) [19] algorithms are used, and the structural features (or residue contacts) are far from being fully exploited. In this regard, GAT-GO [20] utilizes the graph attention (GAT) to discern the importance of different residues upon aggregation, but in essence, the attention score is still based purely on residue node features, and edge information is weakly encoded into the model in an unsupervised and implicit manner [21]. In summary, few efforts have been made to explicitly supervise these residue contacts so that to embed the edge information directly into the model. The residue contacts, as direct reflection of protein structures, happens to be the most crucial feature for function prediction. To address this issue, we propose **SuperEdgeGO**, where we introduce the **Super**vision of **Edge**s upon protein graph learning for better prediction of **G**ene **O**ncology terms. A supervised graph attention mechanism is adopted to encode the residue contacts explicitly in the protein representation, thereby enhancing the model performance. Comprehensive experiments on benchmark datasets demonstrate the superiority of SuperEdgeGO over current state-of-the-art methods.

## Results

### The framework of SuperEdgeGO

**Fig 1:**
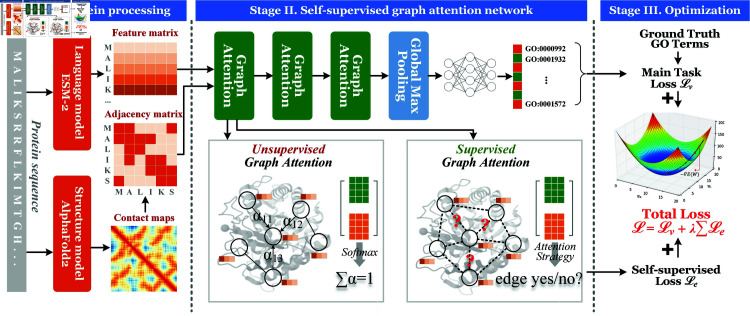
The overall architecture of SuperEdgeGO. **Stage I.** The input protein sequence is first sent to the protein language model ESM-2 to generate the feature matrix, and to the protein structure model AlphaFold2 to predict structures, which is eventually processed as the adjacency matrix. **Stage II.** The two matrices are fed into the model that consists of three graph attention layers, a pooling layer, and a fully-connected classifier. Particularly, the graph attention layer contains both unsupervised and supervised attention modules. **Stage III.** The model is optimized via minimizing two losses, namely the main task loss ${ℒ}_{V}$ arising from the wrong prediction of GO terms, and the self-supervised loss ${ℒ}_{e}$ coming from the deviation of attention scores from the binary label indicating the presence of edges.

The overall framework of SuperEdgeGO is illustrated in Fig 1. It adopts an end-to-end architecture that takes a protein sequence along with its corresponding AlphaFold2-predicted structure as inputs, and outputs a group of GO terms as function prediction results. As shown in the figure, SuperEdgeGO handles proteins in three stages, including protein processing as the first stage, self-supervised graph attention as the second stage, and finally the model optimization. The first stage is basically the process of describing a protein as a graph to be fed into the model. The construction process of the protein graph, including the adjacency matrix (i.e., graph edges) from contact maps and the feature matrix (i.e., residue node features) from ESM-2, is detailed in the Method section. The second stage depicts the model network, with the graph attention layer as the core part. Particularly, each graph attention layer contains an unsupervised attention module and a supervised attention module. Finally, the model is optimized via a joint loss function of the main task and the edge supervision.

### Datasets

To ensure a fair comparison of the model performance, we used the same benchmark dataset as the baseline methods [18], which contains 20,504 human protein data. Briefly, AlphaFold2-predicted protein structures [22] were obtained as the structural information of protein samples, while their corresponding function labels were collected from the gene ontology (GO) annotation labels, aka GO terms. All the samples were divided into training, validation, and test sets in a ratio of 8:1:1. As for the data label, GO terms provide a standardized way to describe the functions of genes and their products (i.e., proteins in our case), and they are organized into three categories to describe the functions of a protein, namely molecular function (MF), biological process (BP), and cellular component (CC). Therefore, the benchmark dataset in this study contains three subsets, namely MF (273 classes), BP (809 classes), and CC (307 classes). Note that rare GO terms appearing less than a certain threshold were filtered out to reduce label sparsity. The model performance was always evaluated across all three GO categories.

### Evaluation metrics

Three commonly used metrics in the protein function prediction task are used to assess the performance of our model, including the protein-centric Fmax, the AUPR (i.e., Area Under the Precision-Recall curves), and the AUC.

Fmax represents the maximum F1 score for all possible threshold values, which is defined as follows:

$$pr(t)=\frac{\sum_{i}\sum_{f}1(f\in {\hat{Y}}_{p,i}^{c}(t)\cap f\in Y_{p,i}^{c})}{\sum_{i}\sum_{f}1\left(f\in {\hat{Y}}_{p,i}^{c}(t)\right)}$$ (1)

$$rc(t)=\frac{\sum_{i}\sum_{f}1\left(f\in {\hat{Y}}_{p,i}^{c}(t)\cap f\in Y_{p,i}^{c}\right)}{\sum_{i}\sum_{f}1\left(f\in Y_{p,i}^{c}\right)}$$ (2)

$$Fmax=\max_{t}\left\{2\times \frac{pr(t)\times rc(t)}{pr(t)+rc(t)}\right\}$$ (3)

where *pr*(*t*) and *rc*(*t*) indicate precision and recall parameterized by threshold t, 1(·) represent the indicator function, f denotes a GO term, ${\hat{Y}}_{p,i}^{c}(t)$ refers to the predicted GO term sets for protein i under the threshold t, and $Y_{p,i}^{c}$ represents the ground truth GO term labels.

Next, for the m-AUPR, it is calculated using the following equations:

$$pr_{f}(t)=\frac{\sum_{i}1(f\in {\hat{Y}}_{p,i}^{c}(t)\cap f\in Y_{p,i}^{c})}{\sum_{i}1\left(f\in {\hat{Y}}_{p,i}^{c}(t)\right)}$$ (4)

$$rc_{f}(t)=\frac{\sum_{i}1(f\in {\hat{Y}}_{p,i}^{c}(t)\cap f\in Y_{p,i}^{c})}{\sum_{i}1(f\in Y_{p,i}^{c})}$$ (5)

$$AUPR=\sum_{t}\left(rc(t)-rc(t-1)\right)\times pr(t)$$ (6)

For a single GO term f, its precision and recall are denoted by *pr*_*f*_(*t*) and *rc*_*f*_(*t*) that parameterized by threshold t. After that, AUPR is calculated globally by taking into account each element of the label indicator matrix as a label.

Finally, AUC represents the area under the ROC curve and is a commonly used metric for unbalanced dataset. The ROC curve is formed by connecting points on axes that display true positive rates (TPR) and false positive rates (FPR) at different thresholds. Then, the area of the coverage area formed by the curve and the x-axis is used as the AUC value of the function label. The AUC formula can be expressed as follows:

$$AUC=\int_{0}^{1}TPR(FPR)d(FPR)$$ (7)

### Hyperparameter settings

The architecture of SuperEdgeGO contains several critical hyperparameters to be optimized before used for training. Since there are too many metrics, i.e., three GO categories with each of them owing three metrics, we picked the *Fmax* of MF-GO as the sole one (except for *the attention strategy* considering its importance in SuperEdgeGO) to fine-tune the parameters to avoid excessive computations. The validation set is used in this phase. All these hyperparameters along with their ranges are listed in Table 1.

**Table 1:** Range of hyperparameter comparison experiments.

Hyperparameter	Range
Attention strategy	AD, DP, MX, SD
Edge sampling rates (*P*_*n*_, *P*_*e*_)	0.1, 0.2, 0.3, …, 1.0
Balancing coefficient ($\lambda_{E}$)	0.001, 0.01, 0.1, 1, 10, 100, 1000
Dropout rate	0, 0.2, 0.4, 0.6, 0.8

The most important hyperparameter to be determined is the attention strategy. As detailed in section *supervised graph attention module*, four candidate strategies including additive attention (AD), dot-product attention (DP), scaled dot-product attention (SD), and mixed attention (MX), are prepared. Experimental results presented in Table 2 suggest that choosing MX as the supervised attention strategy brings the best results in terms of all the metrics in all three categories.

**Table 2:** Performance of SuperEdgeGO under the four supervised attention strategies.

Attention strategies	BP-GO			CC-GO			MF-GO		
	Fmax	AUC	AUPR	Fmax	AUC	AUPR	Fmax	AUC	AUPR
SuperEdgeGO_*AD*_	0.541	0.888	0.577	0.695	0.950	0.763	0.788	0.965	0.841
SuperEdgeGO_*DP*_	0.458	0.856	0.468	0.643	0.919	0.691	0.788	0.968	0.840
SuperEdgeGO_*SD*_	0.545	0.890	0.581	0.696	0.950	0.762	0.793	0.963	0.842
SuperEdgeGO_*MX*_	**0.552**	**0.891**	**0.586**	**0.699**	**0.953**	**0.766**	**0.797**	**0.969**	**0.847**

**Fig 2:**
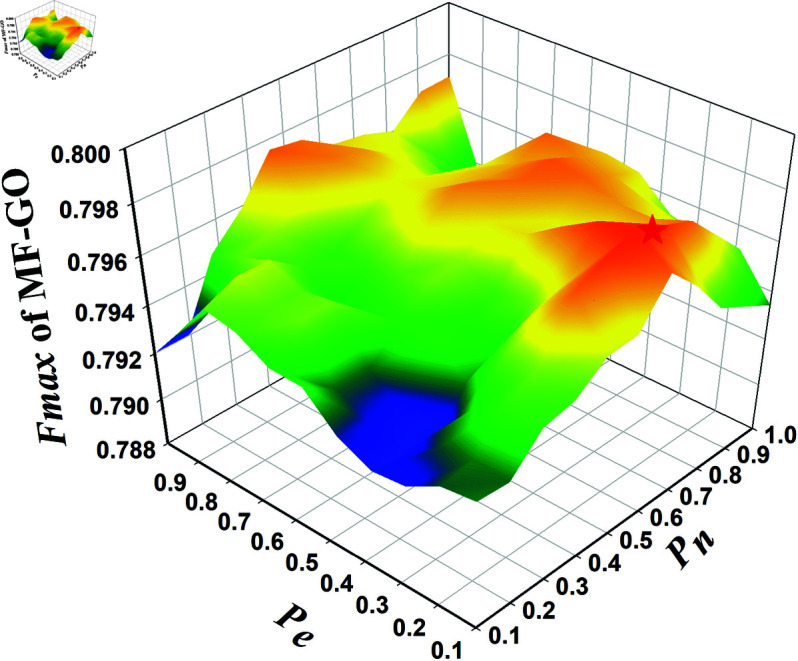
$P_{n}$-$P_{e}$ three-dimensional diagram.

Next, The edge sampling rates *P*_*n*_, *P*_*e*_ are a pair of hyperparameters indicating the number of samples in the edge self-supervision task. Specifically, *P*_*n*_ denotes the negative sampling ratio relative to the number of edges in a protein graph. The negative sampling operation is introduced so that the model is capable of modeling sparse protein graphs while maintaining efficiency. *P*_*e*_ is the overall edge sampling ratio upon training for a regularization effect from randomness. the selection of sampling rates was practically determined through a systematic grid search on the validation set. As illustrated in Fig 2 in the main manuscript, the combination of *P*_*n*_ = 0.6 and *P*_*e*_ = 0.2 achieved the highest Fmax score for MF-GO. Intuitively, adopting *P*_*n*_ = 0.6 enables the inclusion of a reasonable proportion of negative samples, so that the data imbalance in sparse protein graphs can be avoided. In addition, *P*_*e*_ = 0.2 is an explicit regularization operation—by picking only 20% of all samples, the training process is free of overfitting risks.

**Fig 3:**
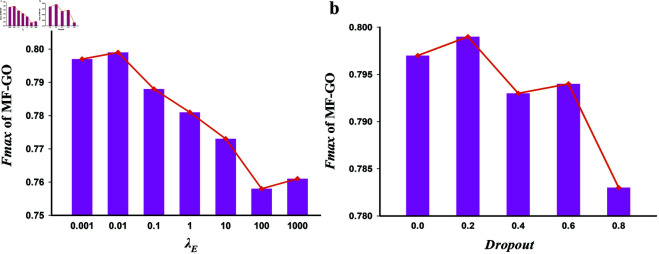
Model performance of different hyperparameter settings. (a) The model achieves its optimal results when $\lambda_{E}$=0.01; (b) The model achieves its optimal results when the dropout rate is set to 0.2.

$\lambda_{E}$ is another critical hyperparameter that balances the loss of the main and the self-supervision task. We tested a series of scaling values from 10^−3^ to 10^3^. As shown in Fig 3, the model yields its best results when $\lambda_{E}$ is set to 0.01.

Finally, the dropout rate is introduced in the fully connected layers indicating the dropping ratio of neurons. It is a simple but effective hyperparameter to prevent overfit. According to the experimental results illustrated in Fig 3, the model achieves its optimal results when dropout is set to 0.2.

### Computational cost analysis

**Fig 4:**
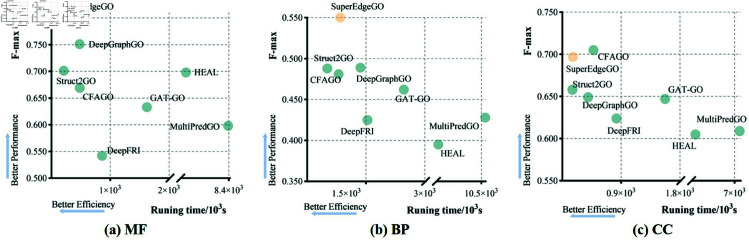
The execution time of SuperEdgeGO and other baseline methods on the (a) MF-GO terms, (b) BP-GO terms, and (c) CC-GO terms of the *Human* dataset. Note that the evaluation was conducted based on NVIDIA GeForce RTX 4090 and may vary depending on the experimental settings.

Despite introducing the edge supervision, SuperEdgeGO remains a highly efficient model. To illustrate this, we plot the model performance of different methods in terms of the F-max metric against their running time in Fig 4, where models closer to the top-left corner are considered superior. It can be observed that SuperEdgeGO is highly efficient among the baseline models while retaining good performance.

### Performance comparison with state-of-the-art approaches

To demonstrate the superiority of SuperEdgeGO over previous methods, we evaluated it on the test set and compared the results with those of some cutting-edge models as follows. The evaluation results are listed in Table 3.

- *Naïve* [7]. The Naïve method is a simple algorithm for function prediction. It predicts the function of a protein based on the frequency of the occurrence of GO terms, regardless of the complexity of the protein sequence or structure.
- *BLAST* [8]. BLAST is a protein function prediction technique based on sequence similarity, which utilizes a dynamic programming algorithm to compare protein sequences and infer functions based on the similarity between sequences.
- *DeepGO* [9]. DeepGO is a deep learning approach that combines protein sequence information extracted by convolutional neural network (CNN) and the macroscopic semantic features from protein-protein interaction (PPI) networks for function prediction.
- *DeepGOA* [10]. DeepGOA applies the graph convolutional network (GCN) to GO-terms hierarchy to acquire knowledge-guided predictions. Meanwhile, CNN is used to extract features from protein sequences.
- *DeepFRI* [5]. DeepFRI pioneeringly introduces the protein-level GCN to the protein function prediction. Proteins are represented as graphs via contact maps, and language models are used to embed the residues.
- *GAT-GO* [20]. GAT-GO introduces the graph attention network (GAT) to replace GCN when dealing with the protein graphs. Note that the attention in GAT-GO is a type of unsupervised attention.
- *Struct2GO* [18]. Struct2GO is a most recent model of protein function prediction. It introduces pretraining techniques on both sequences and graphs, and substring and subgraph are extracted and fused to generate protein representations.

**Table 3:** Performance comparison of SuperEdgeGO and other baseline methods on the *Human* dataset.

Model	BP-GO			CC-GO			MF-GO		
	Fmax	AUC	AUPR	Fmax	AUC	AUPR	Fmax	AUC	AUPR
Naïve	0.347	0.501	0.568	0.571	0.477	0.372	0.336	0.498	0.532
BLAST	0.339	0.577	0.489	0.441	0.563	0.269	0.411	0.623	0.461
DeepGO	0.327	0.639	0.571	0.589	0.695	0.448	0.404	0.760	0.625
DeepGOA	0.385	0.698	0.622	0.629	0.757	0.500	0.477	0.820	0.710
DeepFRI	0.425	0.732	0.635	0.624	0.779	0.641	0.542	0.881	0.763
GAT-GO	0.462	0.586	0.512	0.647	0.831	0.681	0.633	0.912	0.776
Struct2GO	0.481	0.873	0.601	0.658	0.942	0.703	0.701	0.969	0.736
**SuperEdgeGO**	**0.550**	**0.892**	0.586	**0.697**	**0.953**	**0.766**	**0.801**	**0.970**	**0.848**

*Optimal results are shown **in bold**, and sub-optimal results are indicated by underlining.*

According to the evaluation results, SuperEdgeGO achieved state-of-the-art performances in 8 out of 9 metrics except slightly inferior on the AUPR of BP-GO. Among all the three GO categories, SuperEdgeGO gains not only the best results but also the most improvements in MF-GO, with a Fmax of up to 0.801 that significantly higher than the suboptimal value 0.701 achieved by Struct2GO. This is in accordance with the biological intuition; for example, adjacent residues (which are locally contacted) can form pockets thus performing molecular functions like interacting with other proteins or small molecules. As for the rest two categories, SuperEdgeGO also showed tangible improvement, particularly in terms of Fmax (0.550 vs. 0.481 for BP, 0.697 vs. 0.658 for CC). While SuperEdgeGO’s AUPR for BP-GO (0.586) is marginally lower than Struct2GO’s (0.601), it achieves superior Fmax (0.550 vs. 0.481) and AUC (0.892 vs. 0.873). This aligns with the Fmax-centric evaluation protocol in the PFP literature [7,18], prioritizing the balance of precision and recall at optimal thresholds. These results suggest that SuperEdgeGO is an effective tool for predicting all types of protein functions.

In this study, in order to validate the generalizability of the model in protein function prediction, species from different taxonomic categories were selected for cross-species testing, covering *S. cerevisiae* (Saccharomyces cerevisiae), *E. coli* (Escherichia coli), *fruit fly* (Fruit fly), and *rat* (Rattus norvegicus). The results of the experiments are shown in Tables 4, 5, and 6.

**Table 4:** Performance comparison of SuperEdgeGO and other baseline methods on the MF-GO category of the cross-species dataset.

Species	Method	Fmax	AUC	AUPR
*S.cerevisiae*	DeepFRI	0.723	0.891	0.749
	GAT-GO	0.685	0.902	0.733
	**SuperEdgeGO**	**0.763**	**0.953**	**0.795**
*E.coli*	DeepFRI	0.695	0.882	0.722
	GAT-GO	0.681	0.898	0.723
	**SuperEdgeGO**	**0.716**	**0.941**	**0.756**
*Fruit Fly*	DeepFRI	0.731	0.894	0.768
	GAT-GO	0.697	0.887	0.750
	**SuperEdgeGO**	**0.735**	**0.954**	**0.776**
*Rat*	DeepFRI	**0.709**	0.888	0.732
	GAT-GO	0.698	0.895	**0.740**
	**SuperEdgeGO**	0.685	**0.964**	0.721

**Table 5:** Performance comparison of SuperEdgeGO and other baseline methods on the BP-GO category of the cross-species dataset.

Species	Method	Fmax	AUC	AUPR
*S.cerevisiae*	DeepFRI	0.500	0.705	0.478
	GAT-GO	0.501	0.623	0.497
	**SuperEdgeGO**	**0.610**	**0.855**	**0.661**
*E.coli*	DeepFRI	0.485	0.656	0.453
	GAT-GO	0.499	0.677	0.484
	**SuperEdgeGO**	**0.580**	**0.832**	**0.586**
*Fruit Fly*	DeepFRI	0.440	0.674	0.420
	GAT-GO	0.449	0.658	0.441
	**SuperEdgeGO**	**0.475**	**0.913**	**0.468**
*Rat*	DeepFRI	0.375	0.715	0.370
	GAT-GO	0.389	0.728	0.358
	**SuperEdgeGO**	**0.413**	**0.906**	**0.397**

**Table 6:** Performance comparison of SuperEdgeGO and other baseline methods on the CC-GO category of the cross-species dataset.

Species	Method	Fmax	AUC	AUPR
*S.cerevisiae*	DeepFRI	0.676	0.782	0.718
	GAT-GO	0.684	0.805	0.743
	**SuperEdgeGO**	**0.715**	**0.912**	**0.792**
*E.coli*	DeepFRI	0.726	0.812	0.776
	GAT-GO	0.707	0.834	0.763
	**SuperEdgeGO**	**0.791**	**0.960**	**0.858**
*Fruit Fly*	DeepFRI	0.667	0.755	0.697
	GAT-GO	0.664	0.771	0.710
	**SuperEdgeGO**	**0.689**	**0.863**	**0.717**
*Rat*	DeepFRI	0.582	0.798	0.596
	GAT-GO	**0.592**	0.815	0.626
	**SuperEdgeGO**	0.588	**0.950**	**0.627**

The experimental results show that the model is able to identify key features related to protein function more accurately and make effective predictions in the task of protein function prediction in these species.

### Ablation study

We then performed ablation experiments to evaluate the influence of two types of attentions on the model performance. Specifically, we modified SuperEdgeGO to generate three model variants as follows:

- SuperEdgeGO without supervised graph attention (*w/o supv. attn.*): The additional loss item ${ℒ}_{E}$ responsible for the self-supervision on residue contacts is removed. The model degenerates into ordinary GAT with only unsupervised attention upon aggregation.
- SuperEdgeGO without unsupervised graph attention (*w/o unsupv. attn.*): The supervised attention loss ${ℒ}_{E}$ is kept, but the GAT is replaced to GCN that contains no attention coefficients upon aggregation.
- SuperEdgeGO without both attentions (*w/o both attn.*): Both attention types are removed. The model degenerates into GCN in this condition.

We tested these variants on the identical test set as used for the intact architecture. Experimental results are shown in Table 7. It can be interpreted from these results that, both unsupervised and supervised attentions play their roles in improving the model performance, and combining them results in even better results. When eliminating the supervised attention (*w/o supv. attn.*), the model performance dropped in all the three categories in terms of all metrics.

**Table 7:** Ablation experimental results on the human protein dataset.

Model variant	BP-GO			CC-GO			MF-GO		
	Fmax	AUC	AUPR	Fmax	AUC	AUPR	Fmax	AUC	AUPR
*w/o both attn.*	0.530	0.879	0.567	0.690	0.949	0.759	0.780	0.960	0.831
*w/o unsupv. attn.*	0.532	0.888	0.566	0.691	0.951	0.762	0.783	0.965	0.836
*w/o supv. attn.*	0.531	0.881	0.569	0.693	0.952	0.761	0.786	0.963	0.832
*Full*	**0.550**	**0.892**	**0.586**	**0.697**	**0.953**	**0.766**	**0.801**	**0.970**	**0.848**

## Discussion

Converting protein molecules into graph representations using inter-residue contact information has inspired lots of graph-based methods for protein function prediction [23]. However, most existing approaches put efforts in enriching the residue representation while ignoring the latent topology of the residue contacts. The proposed SuperEdgeGO demonstrates that explicit supervision of edge information in protein graphs significantly enhances protein function prediction across all three GO categories. By integrating a supervised attention mechanism to encode residue contacts directly into graph representations, the model achieves state-of-the-art performance, particularly in Molecular Function (MF) prediction, where Fmax improves by 10% over the suboptimal method on the benchmark Human dataset. This underscores the critical role of residue contact features-direct reflections of protein structural topology-in determining functional properties. Further extension to the cross-species dataset also shows superior performance of SuperEdgeGO over conventional graph models, demonstrating the effectiveness and generalizability of the edge supervision design. On the other hand, removing the devised edge supervision attention leads to apparent performance drop, as evidenced by the ablation analysis.

These findings challenge the prevailing assumption in existing graph-based methods that node-centric feature aggregation (e.g., via GCN or GAT) sufficiently captures structural determinants of function. Traditional approaches often treat edge information as passive conduits for message passing, neglecting their intrinsic biological significance. SuperEdgeGO’s edge-supervised paradigm establishes that explicit supervision of residue contacts, guided by their physical existence in contact maps, provides a more biologically grounded representation. This shifts the focus from purely node-centric optimization to a holistic integration of both nodes and edges, aligning computational models more closely with structural biology principles.

The success of edge supervision may extend beyond protein function prediction in the future. For instance, tasks such as drug-target affinity prediction or protein-protein interaction analysis, which also rely on structural insights, could benefit from similar edge-supervised strategies. Another direction that future work should prioritize is the potential impact of using the AlphaFold2-predicted structures rather than the experimentally resolved structures. Although we have previously shown that the predicted structures are comparable to real structures in terms of protein function prediction [24], whether supervision of these predicted edges differs from that of real edges remains to be investigated.

In conclusion, SuperEdgeGO is a new graph-based model for annotating protein functions automatically and efficiently. Comprehensive experiments on benchmark datasets demonstrated that SuperEdgeGO obtained state-of-the-art performance in all three function categories. Ablation analysis further proved the independent contribution of the devised supervised attention module. SuperEdgeGO redefines the role of edges in protein graph representation learning, offering a paradigm shift toward structure-aware supervision. Its success underscores the untapped potential of edge-centric strategies in computational biology, paving the way for more nuanced and accurate models in protein science and beyond.

## Methods

### Problem statement

Given a protein sample *p*, the problem of protein function prediction is a multi-label prediction task that aims to predict a group of function labels represented by multiple GO terms $Y_{p}^{c}=\{y_{p,1}^{c},y_{p,2}^{c},\cdots ,y_{p,N}^{c}\}$, where *c* denotes one of the three broad categories of GO terms (i.e., c∈{MF,BP,CC}), and *N* represents the number of GO terms under the category *c*. Our task is to train a model that takes the protein contact map (or protein graph) ${𝒢}_{p}=\{V_{p},E_{p}\}$ as input, learn an effective representation of the protein, and finally predict its corresponding functional GO terms ${\hat{Y}}_{p}^{c}=\left\{{\hat{y}}_{p,1}^{c},{\hat{y}}_{p,2}^{c},\cdots ,{\hat{y}}_{p,\hat{N}}^{c}\right\}$. Note that each model is specialized for only one category, and therefore there are three models to be trained, i.e., ${\text{Model}}^{\text{MF}}$, ${\text{Model}}^{\text{BP}}$, ${\text{Model}}^{\text{CC}}$.

### The construction of protein graph

In the computational perspective, a protein can be treated as a 2-D graph if being converted to a contact map among residues. In this protein graph denoted as ${𝒢}_{p}=\{V_{p},E_{p}\}$, the node set $V_{p}$ represents all amino acid residues in the protein, while edges *E*_*p*_ are generated via measuring the contact distance between the Cα atoms of two residues. The two residue nodes are connected only if their contact distance is less than a certain threshold. In this study, the threshold is set to 10 Å, which is in consistent with previous work [18]. Note that due to the incomplete experimental-solved structures of the proteins to be predicted, all the structures used in this study were predicted by AlphaFold2 and were collected from [25]. According to our previous preliminary study, the AlphaFold2-predicted structures have been proven to be comparable to real structures in the protein function prediction task.

After the construction of the graph, we next embedded the residue nodes with proper features. Traditional embedding approaches like one-hot encoding scheme based on the amino acid types cannot capture the differences between amino acids of the same type but in different positions, neither the inter-residue relationships. Instead, we used the pretrained protein language model, named ESM-2 (Evolutionary Scale Modeling $2^{nd}$ generation) [26], to generate the initial embedding of residues in a protein. For a protein with $N_{p}$ residues (i.e., $N_{p}=\left|V_{p}\right|$), the ESM-2 language model takes the protein sequence as inputs and generates a feature matrix ${𝐗}_{p}\in {ℛ}^{N_{p}\times D_{p}}$ for the protein, where $D_{p}$ represents the feature dimension of each residue, which are 1280 according to ESM-2. The feature matrix, along with the adjacency matrix ${𝐀}_{p}\in {ℛ}^{{𝐍}_{p}\times {𝐍}_{p}}$, act as initial graph representation and are to be further refined via SuperEdgeGO.

### Unsupervised graph attention module

For the unsupervised graph attention, we adopt a standard graph attention layer (GAT) [27] to update node features. GAT can be treated as a message passing network with adaptive attention weights when aggregating neighboring node features. In our case, it takes the features ${𝐇}_{p}^{l}\in {ℛ}^{{𝐍}_{p}\times F^{l}}$ of a protein graph p in a hidden layer l as inputs, and outputs the updated features ${𝐇}_{p}^{l}\in {ℛ}^{{𝐍}_{p}\times F^{l+1}}$. ${\text{F}}^{\text{l}}$ and *F*^*l* + 1^ denote the feature dimension of a node in the l-th and the subsequent hidden layers, and the initial features ${𝐇}_{p}^{0}$ is the feature matrix ${𝐗}_{p}\in {ℛ}^{{𝐍}_{p}\times {𝐃}_{p}}$ just described in the last section (i.e., $D_{p}=F^{0}=1280$).

To update the feature of a node $h_{i}^{l}$, GAT aggregates all the neighboring nodes of node i as follows:

$${𝐡}_{i}^{l+1}=\sigma \left(\sum_{j\in {𝒩}_{i}\cup \{i\}}\alpha_{ij}{Wh}_{j}^{l}\right)$$ (8)

where ${𝐡}_{i}^{l}\in {ℛ}^{F^{l}}$ is a row vector in ${𝐇}_{p}^{l}$, j is the index of neighboring nodes, σ(·) is the sigmoid activation, and $𝐖\in {ℛ}^{F^{l}\times F^{l+1}}$ is a shared learnable weight matrix to be trained. $\alpha_{ij}$ here, in particular, is an *unsupervised attention score* denoting the relative importance between node i and j. It is calculated as:

$$\alpha_{ij}={softmax}_{j}\left(LeakyReLU(e_{ij})\right)=\frac{\exp\left(LeakyReLU(e_{ij})\right)}{\sum_{k\in {𝒩}_{i}\cup \{i\}}\exp\left(LeakyReLU(e_{ik})\right)}$$ (9)

where LeakyReLU(·) is used as the activation function. Basically, $\alpha_{ij}$ is a softmax normalization over *e*_*ij*_, which is the attention coefficient between node i and j:

$$e_{ij}={𝐚}^{T}[𝐖{𝐡}_{i}^{l}\|𝐖{𝐡}_{j}^{l}]$$ (10)

where $𝐚\in {ℛ}^{2F^{l+1}}$ is a shared attention operation, ^.T^ represents transposition, and || denotes concatenation. Combining Eqs (8)–(10), the node features can be updated.

### Supervised graph attention module

In a perspective of model training, GAT generates an attention score for each edge in an *unsupervised* way. In this condition, the attention is determined adaptively, depending on not only the similarity between the features of two nodes but also the other neighboring nodes due to the normalization operation. To encode edges (residue contacts) more explicitly, we guide the graph attention in a *supervised* way by training the attention score with a binary label indicating whether the edge exists. We denote this *supervised attention score* as $\varphi_{ij}$ to be different with the aforementioned unsupervised one $\alpha_{ij}$:

$$\varphi_{ij}=P\left((i,j)\in E_{p}\right)=\sigma (e_{ij})$$ (11)

where *e*_*ij*_ is the attention coefficient. Different from Eq (9). in which *e*_*ij*_ is activated via LeakyReLU and to be normalized across all the neighboring nodes, under the supervised setting, *e*_*ij*_ is activated with a sigmoid function σ to be mapped between 0 and 1, which is in turn naturally used as a probability P indicating the existence of the edge between nodes i and j. In other words, $\varphi_{ij}$ is trained to reach 1 if the edge exists between nodes i and j (which means that j is important to i), or to reach 0 otherwise. Through this process, the residue contacts are encoded more straightforwardly and explicitly.

**Fig 5:**
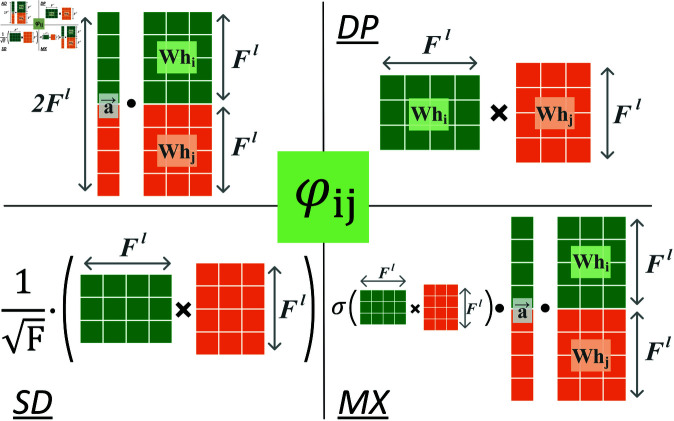
Four strategies to generate the supervised attention score $\varphi_{ij}$.

Inspired by Kim et al. [28], we used four strategies (see Fig 5) to generate $\varphi_{ij}$:

- *Additive attention* (AD). The additive attention follows a standard operation used in GAT, where the two node features are concatenated and mapped to an attention score. It is calculated as:
  $$\varphi_{ij,AD}=\sigma (e_{ij,AD})=\sigma ({𝐚}^{T}[𝐖{𝐡}_{i}∥𝐖{𝐡}_{j}])$$ (12)
  The definition of symbols is the same as in Eq (10).
- *Dot-product attention* (DP). Geometrically, the value of the dot-product is the production of the projections of the two vectors onto the same direction. Therefore, it is a commonly used operation to reflect the similarity between two feature vectors. It is computed as:
  $$\varphi_{ij,DP}=\sigma (e_{ij,DP})=\sigma (({Wh}_{i}{)}^{T}\cdot {Wh}_{j})$$ (13)
- where $𝐖\in {ℛ}^{F^{l}\times F^{l}}$ is a learnable weight matrix shared by the two feature vectors, *e*_*ij*,*DP*_ denotes the attention coefficient between *i* and *j* using the DP strategy.
- *Scaled dot-product attention* (SD). Like in Transformer [29], the dot-product attention is scaled to prevent the dominance of the entire attention by some large values.
  $$\varphi_{ij,SD}=\sigma (e_{ij,SD})=\sigma (e_{ij,DP}/\sqrt{F})$$ (14)
- where *F* denotes the dimension of the node feature vector, *e*_*ij*,*SD*_ denotes the attention coefficient between *i* and *j* using the SD strategy.
- *Mixed attention* (MX). This form of attention mixes AD and DP by converting *e*_*ij*,*DP*_ into a soft gate applied to *e*_*ij*,*AD*_. AD and DP can therefore be used jointly to encode the residue contacts.
  $$\varphi_{ij,MX}=\sigma (e_{ij,MX})=\sigma (e_{ij,AD}\cdot \sigma (e_{ij,DP}))$$ (15)
- where *e*_*ij*,*MX*_ denotes the attention coefficient between *i* and *j* using the MX strategy. $\sigma \left(e_{ij,DP}\right)$ acts as a soft gate to control the feature flow of *e*_*ij*,*AD*_.

The above four strategies for generating the supervised attention score $\varphi_{ij}$ are parallel, which correspond to four variants of SuperEdgeGO. As previously disclosed in Table 2, we evaluated the performance of the four variants by training them individually, and the optimal one was adopted in the architecture.

The supervised and unsupervised attention are fused via sharing the attention coefficients. In detail, the normalized *e*_*ij*_ is used as attention weights in the unsupervised message aggregation, and meantime it is also shared in the supervised branch to predict whether an edge exists. The latter is treated as an auxiliary task and integrated via an additional loss item (see Eq (17)).

### Global aggregation and prediction

To predict the functional GO terms ${\hat{Y}}_{p}^{c}$ of protein p under the category c, all the residue nodes are globally aggregated into a protein-level representation via a max pooling operation, and the protein representation are then sent to the classifier for final multi-label predictions.

$${\hat{Y}}_{p}^{c}=MLP\left(\max_{i=1}^{N_{p}}{𝐡}_{p,i}\right)$$ (16)

where ${𝐡}_{p,i}$ represents the final embedding of a residue node i in protein graph p, and $N_{p}$ indicates the number of residues. MLP(·) represents multilayer perceptron.

### Loss function

One of the key contributions of SuperEdgeGO is that it introduces a supervised graph attention module. Technically, the supervised graph attention belongs to a self-supervision strategy that uses the inherent characteristics (residue contacts in our case) as labels without requiring additional labeling information. Such self-supervised task generates an additional loss, which can be optimized together with the main task loss, therefore forming a multi-task learning paradigm. Formally, the loss function of SuperEdgeGO is computed as follows:

$$ℒ={ℒ}_{v}+\lambda_{E}\cdot \sum_{l=1}^{L}{ℒ}_{E}^{l}$$ (17)

As it can be observed, the loss function has two main components: ${ℒ}_{V}$ is the loss for the main task of the model, which arises from the wrong prediction of GO terms. ${ℒ}_{E}^{l}$ is the loss for self-supervised learning, where l indicates the l-th layer of all L graph attention layers. The loss function for ${ℒ}_{E}^{l}$ takes the form of binary across-entropy and it measures the deviation of the supervised attention score $\alpha_{ij}$ (see Eq (11)) from the binary label indicating the presence of the edge between residue nodes i and j. $\lambda_{E}$ is a balancing coefficient to be determined.

### Data sampling

For the aforementioned edge-supervision task, the positive samples are readily available, which are just all the residue contacts *E*_*p*_ in the protein graph ${𝒢}_{p}$. However, it is not efficient to include all possible negative cases directly due to the generally large number of negative edges (i.e., $(|V_{p}|\times |V_{p}|)\backslash E_{p}$) in some sparse protein graphs. To address this issue, we conducted data sampling when determining the negative sample set $E_{p}^{-}$. This is implemented by introducing a sampling rate $P_{n}\in (0,1]$ on all possible negative samples within the protein graph. After that, another sampling rate $P_{e}\in (0,1]$ over all samples $E_{p}\;\cup \;E_{p}^{-}$ is introduced to act regularization effects and avoid overfitting.
